# Back to the future: A way to increase prosocial behavior

**DOI:** 10.1371/journal.pone.0272340

**Published:** 2022-08-01

**Authors:** Patricia Cernadas Curotto, David Sander, Arnaud d’Argembeau, Olga Klimecki

**Affiliations:** 1 Swiss Center for Affective Sciences, University of Geneva, Geneva, Switzerland; 2 Department of Psychology, University of Geneva, Geneva, Switzerland; 3 Department of Psychology, University of Liège, Liège, Belgium; 4 Faculty of Psychology, Technische Universität Dresden, Dresden, Germany; University of Granada: Universidad de Granada, SPAIN

## Abstract

Previous studies suggest a link between future thinking and prosocial behaviors. However, this association is not fully understood at state and trait level. The present study tested whether a brief future thinking induction promoted helping behavior in an unrelated task. In addition, the relation between mental time travel and prosocial behaviors in daily life was tested with questionnaire data. Forty-eight participants filled in questionnaires and were asked to think about the future for one minute or to name animals for one minute (control condition) before playing the Zurich Prosocial Game (a measure of helping behavior). Results revealed that participants in the future thinking condition helped significantly more than participants in the control condition. Moreover, questionnaire data showed that dispositional and positive orientation toward the future and the past was significantly associated with self-reported prosocial behaviors. The present findings suggest that thinking about the future in general has positive transfer effects on subsequent prosocial behavior and that people who think more about the past or future in a positive way engage more in prosocial behavior.

## Introduction

Anecdotal reports have linked future thinking to increase in prosocial behavior or conflict resolution, even in international conflicts. One example is the Camp David Accords stating the peace agreement between Israel and Egypt in 1978. After several days of negotiations without an agreement at Camp David, the American president Jimmy Carter introduced grandchildren to the discussion. "This was a turning point in the negotiation. Later that day, Begin, Sadat and Carter signed the Camp David Accord." [1]. It is possible that evoking grandchildren shifted the focus from the present to the future, thereby increasing the willingness for compromise and conflict resolution. Does shifting the focus from the present to the future promote constructive and prosocial behaviors? The current study intended to answer this question by testing the impact of future thinking on prosocial behavior.

Shifting the focus from the present to the future involves mental time travel, which has been defined as the ability to mentally project oneself in time either backwards in order to re-live past events or forward to pre-live upcoming events [2]. Even though the phenomenon of mentally reliving past events has received substantial scientific attention [3], there is now a large and growing body of research on future thinking, and in particular on *episodic future thinking*, namely the ability to imagine oneself vividly in a specific future event [4–6]. Extensive research has examined the functions of episodic future thinking in relation to domains such as emotion regulation, intention formation, and planning [7–10].

Evolutionary considerations also argue that mental time travel may guide decision-making and lead to cooperative behaviors by bypassing opportunistic motivation, impulsive choices, and the effects of time discounting [11]. In particular, it has been suggested that episodic future thinking might foster prosocial intentions as well as behaviors [12]. Several studies have provided evidence establishing the link between episodic future thinking and prosocial intentions. More specifically, participants who were asked to imagine themselves helping a person in need expressed more willingness to help this person [13–15]. Furthermore, a recent study has demonstrated that episodic mental simulation (without any temporal locations) of helping events decreased intergroup bias (between Democrats and Republicans), both in the willingness to help and in actual behavior [16]. Similarly, participants who were instructed to adopt a future-oriented perspective (vs. present-oriented perspective) on an interpersonal conflict experienced lower levels of blame toward the partner, more forgiveness, and greater insight [17]. Other researchers have studied other kinds of mental simulations, such as *imagined contact*, the mental representation of a social interaction with outgroup members, and their beneficial impacts on intergroup relations [18, 19]. Indeed, detailed mental simulation of a positive interaction increased positive intentions to engage in outgroup contact [20].

However, the aforementioned studies [13–17, 20] presented a major limitation as the content of the mental simulations was directly related to the outcomes measured. As a result, it might be difficult to disentangle whether the beneficial effect of these mental simulations arose from their future orientation or might be explained by other processes such as *implementation intentions*. Implementation intentions are defined as mental representations linking intention to behaviors: They usually include not only a specific action that someone wants to achieve but also the context in which this action will be performed [21]. Previous research has shown that implementation intentions facilitate the execution of the action and their effectiveness is increased when it is combined with mental imagery [22–25]. Consequently, as mental simulations in previous studies were specific and related to the prosocial behavior measured afterward, implementation intentions may have driven the effect on prosocial behavior.

So far only one study tested the impact of future thinking on altruism using a situation-independent induction of future episodic thinking. In this study, participants who were previously primed to think about the future exhibited a reduction on social discounting compared to those engaged in present thinking [26]. Even though this study provides initial evidence regarding an association between episodic future thinking and prosocial behaviors, the results from this study might suffer from social desirability as the social discounting task was an explicit measure of prosocial behaviors. Future studies in this field may benefit from adopting behavioral tasks to assess prosociality that reduce demand effects, such as the Zurich Prosocial Game (ZPG) [27]. In addition, previous work has mainly focused on the episodic nature of the future thinking (i.e., mental simulation of specific events) and how it relates to prosocial behaviors. Thus, it remains to be tested whether future thinking in general has a causal influence on prosocial behavior.

Likewise, based on the assumption that mental time travel encourages the engagement in prosocial behaviors [11], it also remains to be answered whether people who frequently engage in mental time travel into the past and future also manifest more prosocial behaviors in everyday life. Promising evidence comes from studies arguing that seeing one’s past positively may be a successful method of affective self-regulation, e.g. in order to attenuate aggressive tendencies [28, 29]. Interestingly, a study already showed that participants having pronounced *self-transcendence* (i.e., considering the needs and concerns of others) values were able to perceive near and far future consequences while participants with *self-enhancement* (i.e., optimizing self-interest) goals were only able to take into account near future consequences [30]. However, to date, no study has investigated whether frequent positive mental time travel as a personality trait is related to prosocial behaviors.

Building upon the current literature suggesting that one of the functions of mental time travel may be to favor prosocial behaviors, the aim of the present study was twofold. The first aim was to test the causal relationship between future thinking and prosocial behavior. To this end, we conducted a randomized controlled study to test the extent to which a brief future thinking induction as compared to an active control condition would increase prosocial behavior in the Zurich Prosocial Game [27], a task that measures prosocial behavior in a context that was not directly related to the future thinking task. The second aim was to test whether self-reported positive mental time travel and prosocial behavior in everyday life are correlated.

## Materials and methods

### Participants

The present study was approved by the Ethic Commission of the University of Geneva. A total of 48 participants completed the study. A sensitivity power analysis conducted with G*Power [31] showed that this sample size allows us to detect differences between two independent groups with an effect size of *d* = .83 or bigger, with 80% power. We recruited them using paper advertisements in Geneva. Participants received no remuneration. 24 women and 24 men were randomized to two conditions: future thinking (*Mage* = 24.12, *SD* = 1.75; 12 women) and control condition (*Mage* = 23.04, *SD* = 2.29; 12 women). Participants were either native French-speakers or had a high level of understanding and expression in French. This criterion was specified in the advertisements, and we also asked participants at the very beginning to confirm that they had high level of understanding and expression in French.

S1 Table in S1 File describes demographical data and the groups scores on the Center for Epidemiologic Studies Depression Scale (CES-D) [32], the Balanced Time Perspective Scale (BTPS) [33, 34], the Prosocialness Scale for Adults (PSA) [35], the Self-Report Altruism Scale (SRA) [36], and the Interpersonal Reactivity Index (IRI) [37, 38]. In order to test whether groups differed on these scores independent *t*-tests were performed. These analyses revealed that groups did not differ in any of the demographical data or in questionnaires scores (all *p*_s_ ≥ .055, for further details see the Data Analyses section).

### Experimental design

Participants were randomly assigned to one of two conditions: future thinking or control condition (naming animals). Both interventions took place in person and were implemented prior to playing the Zurich Prosocial Game [27]. Participants in the control condition were asked to name as many animals as possible during one minute and participants in the experimental condition (i.e., future thinking) were instructed to generate as many future events as possible during one minute. This future thinking task was a variant of the Personal Future Task or future fluency task [39, 40] (see Supporting Materials and Methods in S1 File for full instruction and more details). The instructions for the control condition were based on the Semantic Verbal Fluency task, a standard verbal fluency task [41]. The content of the active control condition was selected because it is a typical semantic fluency task and thus both conditions (experimental and control) involved accessing mental contents as fast as possible (for the same duration) but differed in the types of information that needed to be generated (future events vs semantic information).

The instructions for participants in the future condition specified that the future events given by the participants had to take place in the coming year (excluding the next 30 days), that these future events could be positive or negative, specific or general (concrete future project or general expectations for the future). In addition, we asked participants to provide plausible future events, i.e. events that could happen to them in the coming year. While the participant orally enumerated the future events, the experimenter noted the events on paper and recorded them on phone (audio only) to ensure that no future event was missed. After the Zurich Prosocial Game, people in the future thinking condition answered five questions about each future event they had mentioned previously (See Supporting Materials and Methods in S1 File for a full description).

### Measures

#### Zurich prosocial Game

The Zurich Prosocial Game (ZPG) [27] was selected as it provides a measure of prosocial behavior (i.e., helping). In addition, it includes conditions that are designed to measure the impact of reciprocity, costs, and distress on helping behavior. In this game participants move a virtual character (smiley) in a maze to reach treasures and stars in a limited amount of time. Treasures are converted into points. Participants were instructed that the goal of the game was to acquire as many points as possible using treasures and stars. Treasures were worth 50 points and stars which appeared on the paths were worth 20 points, each. Points acquired were not converted to money.

In each round of the game, a different alleged participant was displayed as playing the game at the same time on the screen. Participants were instructed that they would share the screen with a different player for each round and that both players could reach their treasure independently of each other, as each player had two separate paths to choose from. During the game, doors (red or blue) that fell on the paths could block the participant and the other player from advancing to the treasure. Participants were instructed that red keys opened red doors and that blue keys opened blue doors irrespective of the path the doors fell on. Each time participants used their own keys to open the door of another player, this was counted as prosocial behavior. Participants could see the number of doors that were going to fall during the game, the keys they had, the keys of the other player, as well as the time they had left. Each game ended when both players had reached their treasure or when the time was up.

The ZPG included different types of trials in order to test the effect of different factors (reciprocity, distress, cost, and competition) on prosocial behaviors. The ZPG has a total of 11 trials, from which 10 offer the participants the opportunity to help the other, under different conditions that sometimes occur together and are thus not mutually exclusive. To observe the impact of reciprocity on prosocial behavior, four out of ten trials were "Reciprocity" trials in which the participant received help from the other "player" before having the opportunity to reciprocate this help. Conversely, four trials were in the "No Reciprocity" condition, in which the participant did not count on the help from the other. In order to investigate the effects of perceived “Distress” on prosocial behavior, the character of the other "player" began to cry when blocked by a door (visually, and participant heard sounds of crying via headsets) in five trials. There were also trials aiming to evaluate the cost of helping. Among these trials, there were four “high cost” trials where participants pay a price when helping the other player. More precisely, they had to give up their last key of a certain color when helping the other player, while one more door of that color was yet to fall and could thus block the participant from reaching their treasure. In the four “low cost” trials there was no such risk. Finally, three trials aimed to observe whether participants helped rather than increased their own profits (here: points). In these trials, a star appeared at the same time as the other "player" was blocked, leading to a kind of competition between helping the other or scoring more points. These situations were referred to as the "Helping Competition" condition. All trial types were randomized, and each participant completed all ZPG conditions.

#### Questionnaires

In order to assess the tendency to think frequently and positively about one’s past or future, participants completed the Balanced Time Perspective Scale (BTPS) [33, 34]. This scale is composed of 28 items (e.g., "*Creating a positive future is something I often think about*") and comprises two dimensions: half of the items assesses positive past orientation whereas the second half focuses on positive mental simulation into the future. Participants were asked to rate each item on a Likert scale ranging from 1 (*strongly disagree*) to 6 (*strongly agree*).

To assess participants’ prosocial behaviors, we administered the Prosocialness Scale for Adults (PSA) [35] and Self-Report Altruism Scale (SRA) [36]. The PSA includes 16 items related to prosocial attitudes (e.g., "*I share the things I have with my friends*"). Participants were asked to define how well each statement (item) reflects their attitudes on a Likert scale from 1 (*never / almost never true*) to 5 (*almost always / always true*). Regarding the Self-Report Altruism Scale, 20 items describe selfless actions (e.g., "*I have given money to a charity*"). For each item, people were asked to rate how often they perform these actions on a Likert scale of 1 (*never*) to 5 (*very often*).

Finally, we used the Interpersonal Reactivity Index (IRI) [37, 38] to assess four dimensions related to empathy: empathic concern, personal distress, perspective-taking, and fantasy (e.g., "*When I see someone being taken advantage of*, *I feel kind of protective toward them*"). This questionnaire includes 28 items. Participants were asked to rate on a scale from 1 (*did not describe me*) to 7 (*describes me very well*) how well each item describes them.

### Procedure

Participants came to the laboratory individually. After they had read and signed the consent form, they filled in a demographic questionnaire. Then, instructions for the Zurich Prosocial Game (ZPG) [27] were given, followed by questions that were checked by the experimenter who provided additional explanations of the game should this be needed. Depending on the condition of the participant, the Semantic Verbal Fluency task [41] or the Personal Future task [39, 40] were administered. Following this task, participants played the ZPG on a computer. At the end of the ZPG, participants completed the Balanced Time Perspective Scale (BTPS) [33, 34], the Prosocialness Scale for Adults (PSA) [35], the Self-Report Altruism Scale (SRA) [36], and the Interpersonal Reactivity Index (IRI) [37, 38]. These questionnaires were always administered in the same order. Before the experiment ended, we asked participants if they had any comment regarding the experiment. The purpose of this question was to test whether the participants suspected the research aims. As no participant guessed the aims of the study, there was no exclusion. The experiment ended with a debriefing in which we explained that the other players in the ZPG had been alleged and that the aim of the study had been to test the impact of future thinking on prosocial behavior.

### Data analyses

Data were analyzed using R (version 3.5.1) and the packages “psych”, “Hmisc”, and “coin”. Independent *t*-tests revealed that groups did not differ on demographical data, and trait questionnaires (*p*_s_ ≥ .055). However, as the difference between the groups regarding their perspective-taking ability was at trend level (*p* = .055), we first conducted an ANCOVA to test the effect of the condition (future thinking, and control condition) on helping behaviors during the Zurich Prosocial Game using perspective-taking scores as a covariate. This analysis revealed that the covariate was not significant (*p* = .39) and that there was a significant effect of the condition, *F*(1,45) = 6.18, *p* = .017, *η*_*p*_^2^ = .12. Thus, all analyses were run without covariates. The effect of condition (future thinking vs control condition) on helping behaviors was examined with an independent *t*-test. Then, helping behaviors under specific conditions of the ZPG were analyzed using Mann-Whitney U tests as their distributions were not normal. Pearson correlations were also used to calculate the relationships between some of the self-report questionnaires (i.e., Balanced Time Perspective Scale, and Interpersonal Reactivity Index) and the helping behaviors in the ZPG as well as to calculate the relationships between the self-report questionnaires themselves (Balanced Time Perspective Scale, Prosocialness Scale for Adults, Self-Report Altruism Scale, and Interpersonal Reactivity Index). Data are publicly available at https://osf.io/rnpaz/?view_only=5b44698032c14df1b1b29ae249c249e6.

## Results

### Future thinking induction increases prosocial behaviors

**Fig 1:**
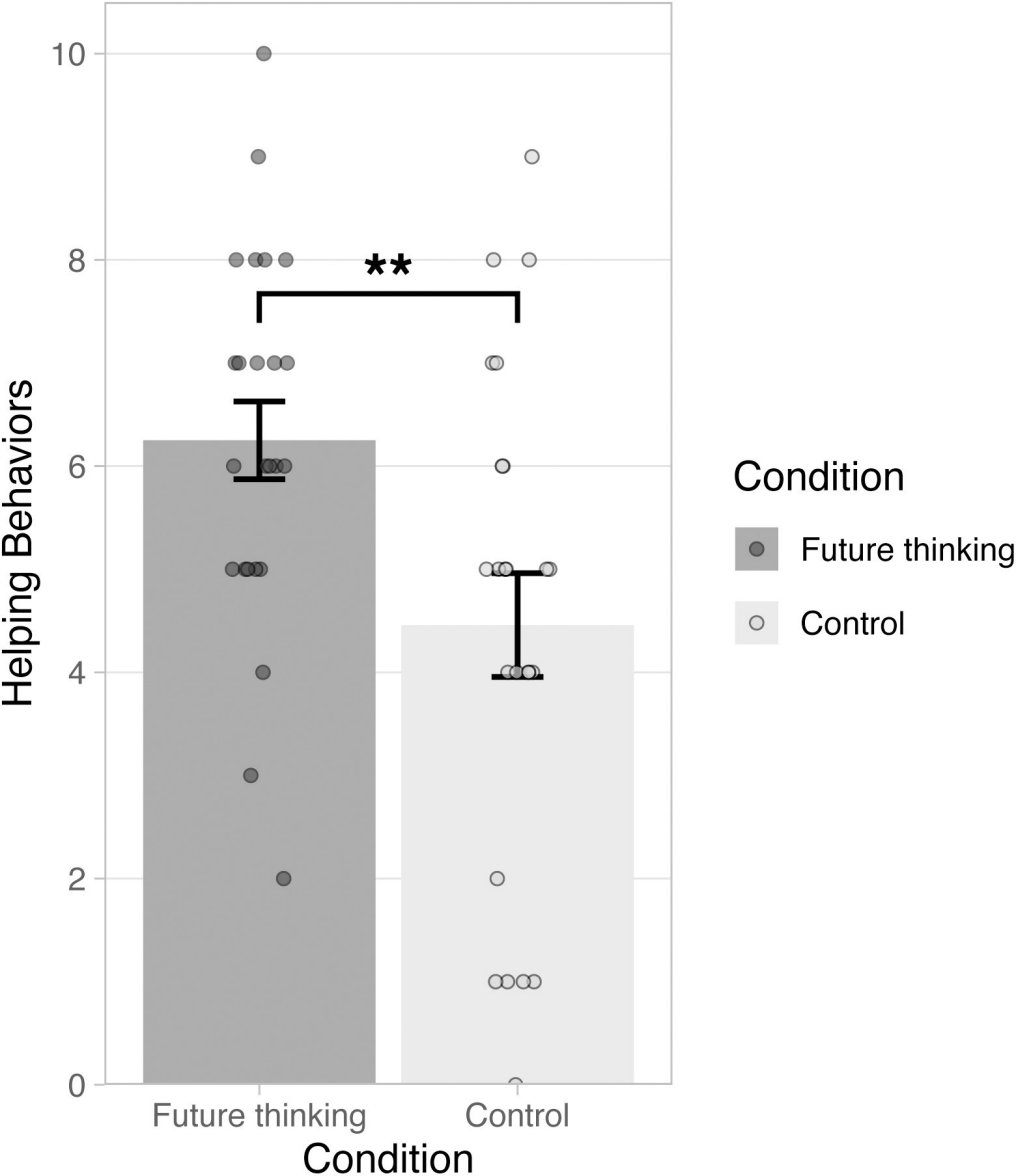
Helping behaviors in the Zurich Prosocial Game as a function of condition (future thinking, control). Errors bars indicate ± 1 *SEM*. Asterisks indicate significant difference between the conditions (*p* < .01).

An independent *t*-test revealed that participants in the future thinking condition (*M* = 6.25, *SD* = 1.85) showed more prosocial behaviors during the Zurich Prosocial Game than participants in the control condition (*M* = 4.46, *SD* = 2.47), *t*(46) = 2.85, *p* = .007, *d* = .82 (Fig 1). As participants could not help in one of the trials of the ZPG, the maximum number of prosocial behaviors (help) was 10.

Differences between the two groups were also observed under some of the ZPG conditions. These differences were tested using Mann-Whitney U tests as the data was not normally distributed. Participants in the future thinking group helped significantly more than participants in the control group in the “Reciprocity”, “Distress”, “Low Cost”, as well as “Helping Competition” conditions (see Table 1).

**Table 1:** Means, standard deviations, Mann-Whitney U tests of prosocial behaviors in different conditions of the Zurich Prosocial Game.

Condition	Future thinking (*n* = 24)		Control (*n* = 24)			
	
	*M*	*SD*	*M*	*SD*	*U*	*p*
Reciprocity	3.33	0.64	2.29	1.49	174	.014*
No Reciprocity	1.79	1.35	1.29	1.16	227	.20
Distress	3.33	0.96	2.25	1.26	152.5	.004**
High Cost	2.42	1.1	1.75	1.26	204.5	.08
Low Cost	2.67	1.13	1.83	1.31	184	.03*
Helping Competition	2.12	0.74	1.42	1.1	184.5	.025*

*Note*. Participants in the future thinking group helped more often under conditions of reciprocity, distress, low cost, and helping competition than participants in the control group. SD: Standard Deviation; df = degrees of freedom

* *p* < .05

** *p* < .01.

### Questionnaire data in relationship with prosocial behavior in the ZPG

To test whether traits assessed by the questionnaires were related to prosocial behaviors in the Zurich Prosocial Game, Pearson correlations were calculated using the whole sample (*N* = 48). These analyses revealed that neither the total sum of the Balanced Time Perspective Scale, nor its subscales (positive past orientation and positive future orientation) significantly correlated with helping behaviors in the ZPG (all *p*_s_ > .16), thus indicating that the disposition to think about positive past or future events is not related to prosocial behavior toward unknown others. Regarding the Interpersonal Reactivity Index (IRI), the analyses revealed a significant correlation between the fantasy subscale and helping behaviors, *r* = .32, *p* = .027. All other correlations were not significant (all other *p*_s_ ≥ .069).

### Relationships between positive mental time travel (trait level) and everyday self-reported prosocial behaviors

**Fig 2:**
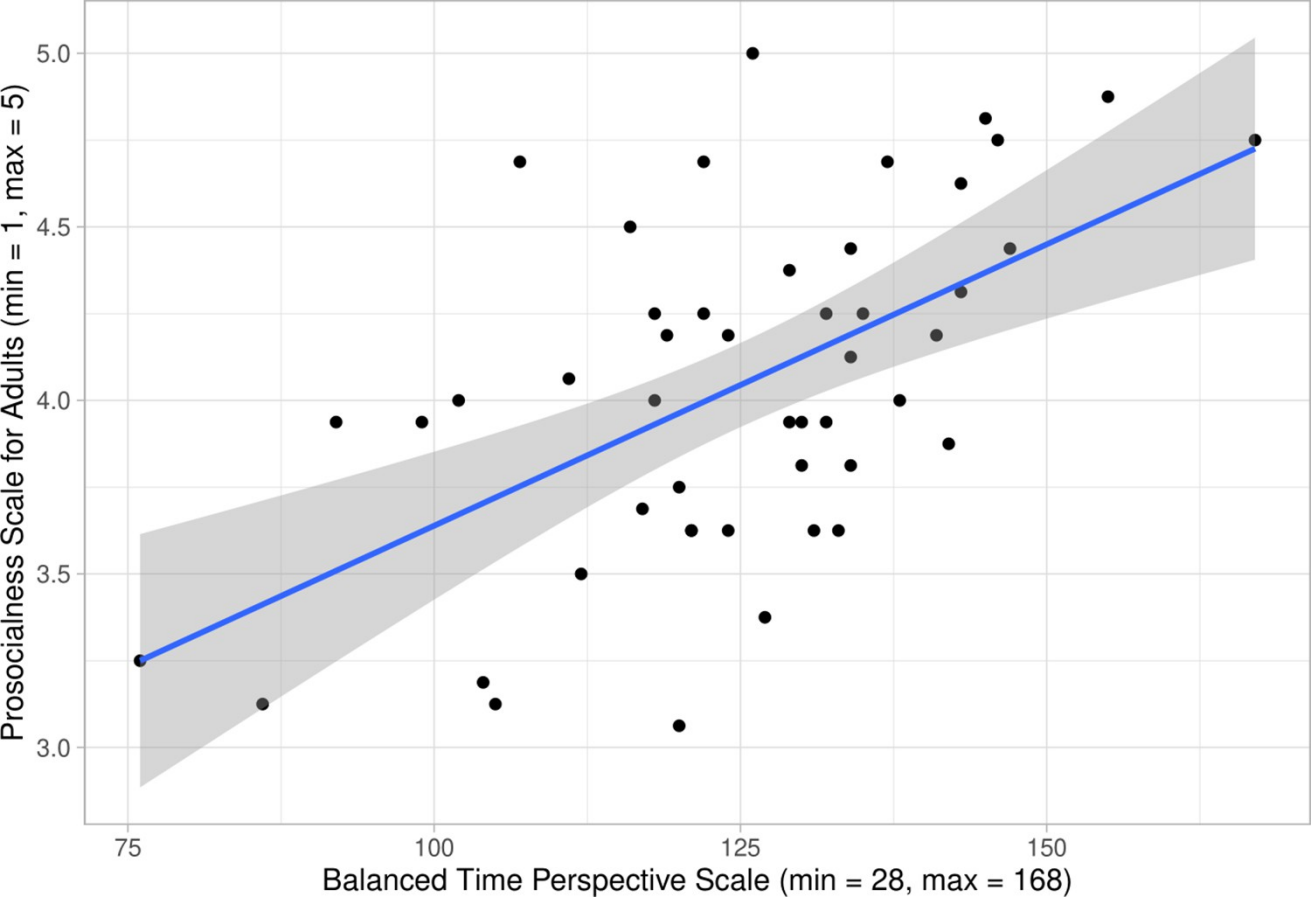
Scatterplot displaying the positive correlations between the total scores of the Balanced Time Perspective Scale and the Prosocialness Scale for Adults.

To test whether there was a relationship between the tendency to think positively about one’s past or one’s future and self-reported prosocial behaviors in everyday life, Pearson correlations were performed between the total score of the Balanced Time Perspective Scale and total scores of the Prosocialness Scale for Adults and Self-Report Altruism Scale on data from all participants. While the correlation between the Balanced Time Perspective Scale and the Self-Report Altruism Scale was not significant, *p* = .27, we found a positive correlation between the Balanced Time Perspective Scale and the Prosocialness Scale for Adults, *r* = .56, *p* < .001 (see Fig 2). Additional Pearson correlations were calculated to investigate other relationships between the different questionnaires. Results are presented in Table 2.

**Table 2:** Intercorrelations between total scores of the questionnaires (N = 48).

Questionnaires	BTPS	PSA	SRA	PT	EC	FS
1. PSA	**.56** ***					
2. SRA	.16	**.32** *				
3. PT	.22	**.49** ***	.07			
4. EC	**.53** ***	**.79** ***	**.41** ***	**.36** *		
5. FS	.22	**.40** **	.20	.12	**.47** **	
6. PD	.17	.19	-.22	-.10	.21	**.34** *

*Note*. BTPS = Balanced Time Perspective Scale; PSA = Prosocialness Scale for Adults; SRA = Self-Report Altruism Scale; PT = Perspective-Taking (from Interpersonal Reactivity Index); EC = Empathic Concern (from Interpersonal Reactivity Index); FS = Fantasy (from Interpersonal Reactivity Index); PD = Personal Distress (from Interpersonal Reactivity Index).

*p < .05.

**p < .01.

***p < .001.

## Discussion

The present study confirmed the main hypothesis that a short induction of future thinking can produce transfer effects by fostering helping behaviors in an unrelated context. This result extends earlier studies which showed a link between mental simulations (imagined contact simulation, and episodic future thinking) related to a certain situation to prosocial intentions or behaviors in these situations [13–17, 20] and studies based on explicit paradigms assessing prosocial behavior [26]. Importantly, the present study extends these findings by showing that a situation-independent and general future thinking induction paradigm can increase prosocial behavior in an implicit task: the Zurich Prosocial Game [27]. In particular, to avoid any social desirability bias, the Zurich Prosocial Game was introduced as a video game in which the goal was to maximize one’s own points and no reference to helping was made in the instructions.

Consequently, we believe that these beneficial effects could extend to other contexts in which prosocial behaviors may be difficult, for instance in real world settings such as intergroup conflicts. Future thinking may counteract one of the major challenges faced by policymakers and researchers when they test interventions in intergroup conflicts: the motivation of the individuals to change their emotions or attitudes toward outgroup members [42, 43]. Indeed, as we saw in this study, the induction of future thinking worked independently of the content of the prospections. In other words, there is no need to mention the outgroup in order to shift individuals time perspective, thus avoiding motivational obstacles. In line with this suggestion, recent research revealed that an episodic simulation induction (unrelated to the subsequent empathy measure) increased subsequent affective empathic responses not only toward ingroup but also toward outgroup members [44].

Importantly, here we also showed, for the first time, preliminary evidence for an association between positive mental time travel and prosocial behaviors in daily life at trait level. More precisely, projecting oneself positively into the future and projecting oneself positively into the past (assessed by two subscales of the Balanced Time Perspective Scale) were associated to Prosocialness Scale for Adults (PSA), but not with the Self-Report Altruism Scale (SRA). One possible explanation might be that these two questionnaires seem to differ on many points and may capture different aspects of prosociality. In both self-reported questionnaires participants rate the frequency with which they perform prosocial behaviors. However, the PSA items address more common situations (e.g., I am available for volunteer activities to help those who are in need) than SRA items (e.g., I have given a stranger a lift in my car). In addition, SRA targets more concrete and specific acts of helping (e.g., I have offered my seat on a bus or train to a stranger who was standing) while PSA items present more general situations without mentioning explicitly ways to help (e.g., I help immediately those who are in need). Thus, by being more generic, PSA items capture several ways to be prosocial while SRA narrows a limited number of prosocial acts. We believe that PSA thus offers more opportunities for reporting prosocial behavior than SRA. Further studies should investigate in more depth what kind of prosocial behaviors are related or facilitated by mental time travel in daily life.

Surprisingly, no association between the disposition to think positively about past or future events and prosocial behaviors assessed by the Zurich Prosocial Game was found. Future research with larger sample sizes and broader measures should examine closely whether mental time travel as a trait predicts actual prosocial behaviors. A remained unanswered question is whether the trait to engage in future thinking per se, as opposed to positive mental time travel is also related to prosocial behavior.

The present randomized controlled study shows causal evidence that thinking about the future in general can promote prosocial behavior in an unrelated context. As this is a novel result, replications from other laboratories, with other paradigms and larger samples are needed to solidify this finding. Besides, a key limitation of this study is that we cannot ensure that the effect found is completely due to a shifting of the focus from the present to the future. Indeed, the two conditions differed in a series of parameters other than the future dimension (e.g., events vs. animals generated, self-related vs. not related). Further work should isolate the specific effect of future thinking by having a control condition that only differs in terms of temporal orientation, for instance remembering past events (concrete or abstract) or thinking about present events [26]. In addition, although the Zurich Prosocial Game was used here to assess helping behaviors with a more ecological measure rather than relying solely on self-reports, some of the trials may disrupt the illusion that the participant is playing with another person. More specifically, in the “Distress” condition, participants can hear the cries of the other player’s character. However, participants cannot influence the cries of their character. Different ecological paradigms should be adopted to capture prosocial behavior in order to establish the causal link between future thinking and prosocial behavior. Finally, the scales used in this study were always presented after the manipulation of future thinking or verbal fluency and after playing the Zurich Prosocial Game. Therefore, the possibility that these manipulations or the game influenced participants’ reports cannot be ruled out. Future work could control for this aspect, for example, by administering the questionnaires prior to the manipulation or by counterbalancing the time of administration of the questionnaires.

## Conclusions

The present study shows that a short induction of general future thinking promotes helping behaviors toward strangers when compared to an active control condition (naming animals). Importantly, the future thinking intervention was situation-independent from the measure of prosocial behaviors, which was carried out with a task aimed at reducing social desirability. The observed findings in the laboratory were mirrored by results on the link between self-reported prosocial behaviors and positive mental time travel in daily life. Participants who reported thinking positively about the future or remembering the past positively reported more prosocial behaviors. Taken together, the present findings suggest that (positive) general future thinking is a way to promote prosocial behaviors.

Looking back to the strategy adopted by the American president Jimmy Carter—discussing about grandchildren while negotiating a peace agreement—may be a powerful way to implicitly induce constructive behaviors through future thinking. Further work is required to establish the efficiency of such short induction of future thinking in intergroup conflicts.

## Supporting information

S1 File Supporting information (including Supporting Materials and Methods, Supporting Results, and Supporting Tables). (PDF)

## References

[1] Fisher R, Shapiro D. Beyond reason: Using emotions as you negotiate. New York: Penguin Group; 2005.

[2] Suddendorf T, Corballis MC. The evolution of foresight: What is mental time travel, and is it unique to humans? Behav Brain Sci. 2007;30:299–351. doi: 10.1017/S0140525X07001975 17963565

[3] Schacter DL, Addis DR, Buckner RL. Remembering the past to imagine the future: The prospective brain. Nat Rev Neurosci. 2007;8:657–61. doi: 10.1038/nrn2213 17700624

[4] Szpunar KK, Spreng RN, Schacter DL. A taxonomy of prospection: Introducing an organizational framework for future-oriented cognition. Proc Natl Acad Sci. 2014;111(52):18414–21. doi: 10.1073/pnas.1417144111 25416592 PMC4284580

[5] Szpunar KK. Episodic future thought: An emerging concept. Perspect Psychol Sci. 2010;5(2):142–62. doi: 10.1177/1745691610362350 26162121

[6] Atance CM, O’Neill DK. Episodic future thinking. Trends in Cognitive Sciences. 2001;5(12):533–539. doi: 10.1016/s1364-6613(00)01804-0 11728911

[7] Schacter DL, Benoit RG, Szpunar KK. Episodic future thinking: mechanisms and functions. Curr Opin Behav Sci. 2017;17:41–50. doi: 10.1016/j.cobeha.2017.06.002 29130061 PMC5675579

[8] D’Argembeau A, Renaud O, Van der Linden M. Frequency, characteristics and functions of future-oriented thoughts in daily life. Appl Cogn Psychol. 2011;25(1):96–103.

[9] Barsics C, Van der Linden M, D’Argembeau A. Frequency, characteristics, and perceived functions of emotional future thinking in daily life. Q J Exp Psychol. 2016;69:217–33. 10.1080/17470218.2015.1051560 26140455

[10] Schacter DL. Adaptive constructive processes and the future of memory. Am Psychol. 2012;67(8):603–13. doi: 10.1037/a0029869 23163437 PMC3815569

[11] Boyer P. Evolutionary economics of mental time travel? Trends Cogn Sci. 2008;12(6):219–24. doi: 10.1016/j.tics.2008.03.003 18468941

[12] Gaesser B. Constructing Memory, Imagination, and Empathy: A Cognitive Neuroscience Perspective. Front Psychol. 2013;3. 10.3389/fpsyg.2012.00576 PMC3579581 23440064

[13] Gaesser B, Schacter DL. Episodic simulation and episodic memory can increase intentions to help others. Proc Natl Acad Sci. 2014;111(12):4415–20. doi: 10.1073/pnas.1402461111 24616532 PMC3970486

[14] Gaesser B, Horn M, Young L. When Can Imagining the Self Increase Willingness to Help Others? Investigating Whether the Self-Referential Nature of Episodic Simulation Fosters Prosociality. Soc Cogn. 2015;33(6):562–84.

[15] Gaesser B, DiBiase HD, Kensinger EA. A role for affect in the link between episodic simulation and prosociality. Memory. 2017;25(8):1052–62. doi: 10.1080/09658211.2016.1254246 27841093

[16] Gaesser B, Shimura Y, Cikara M. Episodic simulation reduces intergroup bias in prosocial intentions and behavior. J Pers Soc Psychol. 2020;118(4):683–705. doi: 10.1037/pspi0000194 31157527

[17] Huynh AC, Yang DYJ, Grossmann I. The Value of Prospective Reasoning for Close Relationships. Soc Psychol Personal Sci. 2016;7(8):893–902.

[18] Miles E, Crisp RJ. A meta-analytic test of the imagined contact hypothesis. Gr Process Intergr Relations. 2014;17(1):3–26.

[19] Crisp RJ, Turner RN. Can Imagined Interactions Produce Positive Perceptions? Reducing Prejudice Through Simulated Social Contact. Am Psychol. 2009;64(4):231–40. doi: 10.1037/a0014718 19449982

[20] Husnu S, Crisp RJ. Elaboration enhances the imagined contact effect. J Exp Soc Psychol. 2010;46:943–50.

[21] Gollwitzer PM. Goal Achievement: The Role of Intentions. Eur Rev Soc Psychol. 1993;4(1):141–85.

[22] Cameron LD, Chan CKY. Designing Health Communications: Harnessing the Power of Affect, Imagery, and Self-Regulation. Soc Personal Psychol Compass. 2008;2(1):262–82.

[23] Gollwitzer PM, Brandstätter V. Implementation Intentions and Effective Goal Pursuit. J Pers Soc Psychol. 1997;73(1):186–99. 10.1037//0022-3514.81.5.946 11708569

[24] Holland RW, Aarts H, Langendam D. Breaking and creating habits on the working floor: A field-experiment on the power of implementation intentions. J Exp Soc Psychol. 2006;42(6):776–83.

[25] Pham LB, Taylor SE. From thought to action: Effects of process- versus outcome-based mental simulations on performance. Personal Soc Psychol Bull. 1999;25(2):250–60.

[26] Yi R, Pickover A, Stuppy-Sullivan AM, Baker S, Landes RD. Impact of episodic thinking on altruism. J Exp Soc Psychol. 2016;65:74–81. doi: 10.1016/j.jesp.2016.03.005 27821875 PMC5096404

[27] Leiberg S, Klimecki O, Singer T. Short-Term Compassion Training Increases Prosocial Behavior in a Newly Developed Prosocial Game. Verdejo García A, editor. PLoS One. 2011;6(3):e17798. doi: 10.1371/journal.pone.0017798 21408020 PMC3052380

[28] Stolarski M, Bitner J, Zimbardo PG. Time perspective, emotional intelligence and discounting of delayed awards. Time Soc. 2011;20(3):346–63.

[29] Stolarski M, Zajenkowski M, Zajenkowska A. Aggressive? from Time to Time… Uncovering the Complex Associations between Time Perspectives and Aggression. Curr Psychol. 2016;35(4):506–15.

[30] Brosch T, Stussi Y, Desrichard O, Sander D. Not my future? Core values and the neural representation of future events. Cogn Affect Behav Neurosci. 2018;18:476–84. doi: 10.3758/s13415-018-0581-9 29557086

[31] Faul F, Erdfelder E, Lang A-G, Buchner A. G*Power 3: A flexible statistical power analysis program for the social, behavioral, and biomedical sciences. Behav Res Methods. 2007;39(2):175–91. doi: 10.3758/bf03193146 17695343

[32] Morin AJS, Moullec G, Maïano C, Layet L, Just JL, Ninot G. Psychometric properties of the Center for Epidemiologic Studies Depression Scale (CES-D) in French clinical and nonclinical adults. Rev Epidemiol Sante Publique. 2011;59(5):327–40. doi: 10.1016/j.respe.2011.03.061 21925817

[33] Barsics C, Rebetez MML, Rochat L, D’Argembeau A, Van der Linden M. A French version of the balanced time perspective scale: Factor structure and relation to cognitive reappraisal. Can J Behav Sci. 2017;49(1):51–7.

[34] Webster JD. A new measure of time perspective: Initial psychometric findings for the balanced time perspective scale (BTPS). Can J Behav Sci. 2011;43(2):111–8.

[35] Caprara GV, Steca P, Zelli A, Capanna C. A New Scale for Measuring Adults’ Prosocialness. Eur J Psychol Assess. 2005;21(2):77–89.

[36] Rushton PJ, Chrisjohn RD, Fekken CG. The altruistic personality and the self-report altruism scale. Pers Individ Dif. 1981;2(4):293–302.

[37] Davis MH. Measuring individual differences in empathy: Evidence for a multidimensional approach. J Pers Soc Psychol. 1983;44(1):113–26.

[38] Gilet A-L, Mella N, Studer J, Grühn D, Labouvie-Vief G. Assessing dispositional empathy in adults: A French validation of the Interpersonal Reactivity Index (IRI). Can J Behav Sci Can des Sci du Comport. 2013;45(1):42–8.

[39] MacLeod AK, Rose GS, Williams JMG. Components of hopelessness about the future in parasuicide. Cognit Ther Res. 1993;17(5):441–55.

[40] MacLeod AK, Byrne A. Anxiety, depression, and the anticipation of future positive and negative experiences. J Abnorm Psychol. 1996;105(2):286–9. doi: 10.1037//0021-843x.105.2.286 8723011

[41] Godefroy O, GREFEX. Fonctions exécutives et pathologies neurologiques et psychiatriques. Evaluation en pratique clinique. Solal. Marseille;

[42] Porat R, Halperin E, Tamir M. What we want is what we get: Group-based emotional preferences and conflict resolution. J Pers Soc Psychol. 2016;110(2):167–90. doi: 10.1037/pspa0000043 26785061

[43] Klimecki OM, Vétois M, Sander D. The impact of empathy and perspective-taking instructions on proponents and opponents of immigration. Humanit Soc Sci Commun. 2020;7(91):1–12.

[44] Vollberg MC, Gaesser B, Cikara M. Activating episodic simulation increases affective empathy. Cognition. 2021;209:104558. doi: 10.1016/j.cognition.2020.104558 33385949

