# Supplementary material for: Back to the future: A way to increase prosocial behavior
Source: PLoS One. 2022 Aug 1;17(8):e0272340. doi: 10.1371/journal.pone.0272340 (PMC9342755; doi:10.1371/journal.pone.0272340)
Supplement: S1 File — (PDF) [file pone.0272340.s001.pdf]

Back to the future: a way to increase prosocial behavior

Patricia Cernadas Curotto[1,2], David Sander[1,2], Arnaud d'Argembeau[1,3], Olga Klimecki[1, 4]

[1] Swiss Center for Affective Sciences, University of Geneva, Geneva, Switzerland

[2] Department of Psychology, University of Geneva, Geneva, Switzerland

[3] Department of Psychology, University of Liège, Liège, Belgium

[4] Faculty of Psychology, Technische Universität Dresden, Dresden, Germany

Supporting Materials and Methods

Participants

Psychology students or participants of psychology courses were excluded. We asked participants whether there were under medication: none of the participant was under medication. Participants completed a series of questionnaires at the very beginning of the experiment. We then tested whether groups differed on prosocial behaviors assessed by the Prosocialness Scale for Adults (PSA) [2] and the Self-Report Altruism Scale (SRA) [3], on empathic traits measured by Interpersonal Reactivity Index (IRI) [4,5] and on the positive disposition to mental time travel captured by the Balanced Time Perspective Scale (BTPS) [6,7]. Moreover, as depressed individuals differ in prosocial responses compared to healthy adults [8], the Center for Epidemiologic Studies Depression Scale (CES-D) [9,10] was used in order to test whether groups differed in depression levels after group allocation. The scores on these questionnaires as well as the demographical data are summarized in S1 Table 1.

S1 Table 1. Independent *t*-tests between groups (Future thinking, Control) on demographical data and questionnaires.

	Future thinking (n = 24)		Control (n = 24)		Group Difference		
	Mean	*SD*	*Mean*	*SD*	*t*	*df*	*p*
**Age**	24.12	1.75	23.04	2.29	1.84	46	.072
**Education level (years)**	17.27	1.59	16.17	2.48	1.84	39.25	.074
**Center for Epidemiologic Studies Depression Scale**	13.29	8.07	11.33	6.66	0.92	46	.36
**Balanced Time Perspective Scale (total)**	122.46	20.99	127.38	12.89	-0.98	38.19	.33
**Past Orientation**	56.21	13.27	58.71	8.88	-0.77	46	.45
**Future Orientation**	66.25	10.17	68.67	6.29	-0.99	38.37	.33

(*Continued*)

S1 Table 1. (Continued)

	Future thinking (n = 24)		Control (n = 24)		Group Difference		
	Mean	*SD*	*Mean*	*SD*	*t*	*df*	*p*
**Prosocialness Scale for Adults**	4.14	0.53	3.95	0.46	1.37	46	.18
**Self-Report Altruism Scale**	57.96	10.67	54.75	8.01	1.18	46	.24
**Interpersonal Reactivity Index** Perspective-Taking	37.67	5.96	33.96	7.02	1.97	46	.055
**Interpersonal Reactivity Index** Empathic Concern	37.12	7.15	35.12	6.22	1.03	46	.31
**Interpersonal Reactivity Index** Fantasy	36.17	7.27	32.92	6.98	1.58	46	.12
**Interpersonal Reactivity Index** Personal Distress	23.58	7.99	23.83	7.68	-0.11	46	.91

Note. As the variances were not equal for the education level, the Balanced Time Perspective Scale (total), and the Future Orientation subscale, Welch's *t*-tests were used for these data. SD: Standard Deviation; df = degrees of freedom

Cover Story

The current study used a cover story to disguise the true purpose of the research. Here participants were told that they would participate in a study on language skills and performance in a video game. In order to reinforce this belief, fictitious goals were written on the consent form but also on several documents related to the experience (questionnaires, and game instructions). We also included at the beginning of the experiment questions about participants' language skills, such as: "*Are you bilingual? If yes, what is your second most commonly spoken language?*" Furthermore, the study was framed as being part of a project bringing together several researchers of different universities across Europe. Therefore, the EU flag was visible on several documents that participants were asked to read. We also emphasized this point orally, for instance, when the game instructions were explained (e.g., "Each new round, you will be randomly connected with other participants in different research institutes across Europe"). At the end of the experiment, the real aims of the present study were communicated to the participants.

Instructions for participants in the future condition

"Now I would like you to imagine for one minute as many events as possible that could happen to you in the next year (excluding the next 30 days).

These events could be positive or negative. They can be specific events that are likely to happen at a specific time in the future, or more general events about your plans and/or expectations for the future. However, avoid events that refer to states of mind (e.g. I will be happy) and prefer concrete events. These events should be plausible and reasonably likely to happen in the coming year. Is this OK? Do you have any questions?

Try to list as many events as possible, bearing in mind that you only have one minute, so do not loose time by describing the events.

When I tell you to start, list as many future events as you can and continue to tell me as many things that might happen in the next year (excluding the next 30 days) until the minute is up. Is that clear? Are you ready?"

The current instructions stated that participants had to exclude future events in the next 30 days to prevent that these events were too close in time to the present. This criterion was adopted based on the *Construal-Level Theory of Psychological Distance* [1], which suggests that mental time travel can be considered as a form of traversing psychological distance, with the self in the moment present as reference point. In addition, this theory also states that different psychological distance dimensions (e.g., in time or in social distance) are interrelated, such that a manipulation of one of these dimensions may also affect the other. Building upon this, we decided to exclude the next 30 days in our future thinking manipulation to avoid events that involve little psychological distance, and thus to maximize the potential influence of our manipulation on psychological social distance.

Additional measures

Mood.

As it has been shown that mood can influence helping behaviors [11], previous research on prosocial behaviors have controlled the mood of participants [12]. Here we also tested whether participants differed in terms of mood before playing the Zurich Prosocial Game using one single item. More precisely, participants rated their mood level on a scale ranging from -3 (*negative mood*) to +3 (*positive mood*). Importantly, participants in the future thinking condition (M = 1.79, SD = 1.25) did not differ from participants in the control condition (M = 1.83, SD = 1.27), $t(46) = -.11$ $p = .91$.

Questionnaire on the future event characteristics.

Participants in the future thinking condition completed additional questions related to the future events that they had named. To this end, the experimenter had copied the future events listed by the participants in the Personal Future Task. Each future event generated in the Personal Future task was evaluated on four phenomenological characteristics: the positive emotions felt, the negative emotions felt, the clarity and sensory richness, and the autonoetic consciousness (i.e., a form of consciousness allowing individuals to apprehend the connection between one's current self and the future or the past). Using a Likert scale from 1 (*not at all*) to 7 (*a lot*) participants evaluated how many positive emotions and negative emotions they felt about the event. A 7-point Likert scale from 1 (*not at all*) to 7 (*extremely*) was used to assess clarity and sensory richness (visual, auditory, olfactory, gustatory and / or tactile sensory details) of the future event. For rating autonoetic consciousness, two items were used ("*you feel that you are actually experiencing the event, as if you were there*" and "*the impression of going into the future and being at that moment when this event would occur*"). Participants rated these two items on a Likert scale in 7 points (from 1 = *not at all* to 7 = *completely*) and these scores were then averaged in order to obtain an index of autonoetic consciousness. The future event was listed at the top of the sheet to prevent the person from confusing the events with each other when rating them. Before the rating of events started, the experimenter made sure that the participant understood each dimension.

Questions about future event listed

Keyword (to recover the event that has been listed):

1. To what extent did you feel positive emotions about this event? Circle the number that best describes your feelings.

From 1 (*not at all*) to 7 (*a lot*)

2. To what extent did you feel negative emotions about this event? Circle the number that best describes your feelings.

From 1 (*not at all*) to 7 (*a lot*)

When you mentally picture this event

3. Your thinking is clear (places, people and objects involved) and includes sensory details (visual, auditory, olfactory, gustatory and/or tactile).

From 1 (*not at all*) to 7 (*extremely*)

4. When thinking about this event, you feel that you are actually experiencing the event, "as if you were there".

From 1 (*not at all*) to 7 (*completely*)

5. When thinking about this event, you have the impression of going into the future and being at that moment when this event would occur.

From 1 (*not at all*) to 7 (*completely*)

Supporting Results

The following analyses included only half of the participants ($n = 24$), the participants in the future thinking condition. On average, participants generated 10.79 ($SD = 2.4$) prospections. Descriptive data on the characteristics of the prospections are presented in S1 Table 2.

S1 Table 2. Means, standard deviations, minimum and maximum of prospection characteristics ($n = 24$)

Characteristics	*M*	*SD*	*Min-Max*
Positive emotions	5.22	0.76	3.19 - 6.38
Negative emotions	2.43	0.84	1 - 4.31
Clarity and sensory richness	3.71	0.8	2.54 - 5.38
Autonoetic consciousness	3.6	0.8	2.55 - 5.59

Note. SD: Standard Deviation

Prospections' characteristics in relation with prosocial behavior in the Zurich Prosocial Game

In order to test whether the characteristics of the prospection were related to helping behavior during the Zurich Prosocial Game, we performed Pearson correlations. In spite of the small sample used ($n = 24$), a marginal trend was found for the correlation between positive emotions and helping, $r = .39$, $p = .062$. None of the other correlations was significant (all other $p_s > .21$). These findings differ from previous studies showing a relationship between vivid mental simulations and prosocial behaviors [13,14]. Our findings might be limited not only by the small sample size but as well as by the limited amount of time that participants had to generate as many prospections as possible, thus preventing them to make elaborated prospections.

References

1. Trope Y, Liberman N. Construal-level theory of psychological distance. Psychol Rev. 2010;117(2):440–63.
2. Caprara GV, Steca P, Zelli A, Capanna C. A New Scale for Measuring Adults' Prosocialness. Eur J Psychol Assess. 2005 Jan;21(2):77–89.
3. Rushton PJ, Chrisjohn RD, Fekken CG. The altruistic personality and the self-report altruism scale. Pers Individ Dif. 1981;2(4):293–302.
4. Davis MH. Measuring individual differences in empathy: Evidence for a multidimensional approach. J Pers Soc Psychol. 1983;44(1):113–26.
5. Gilet A-L, Mella N, Studer J, Grühn D, Labouvie-Vief G. Assessing dispositional empathy in adults: A French validation of the Interpersonal Reactivity Index (IRI). Can J Behav Sci Can des Sci du Comport [Internet]. 2013;45(1):42–8.
6. Barsics C, Rebetez MML, Rochat L, D'Argembeau A, Van der Linden M. A French version of the balanced time perspective scale: Factor structure and relation to cognitive reappraisal. Can J Behav Sci. 2017;49(1):51–7.
7. Webster JD. A new measure of time perspective: Initial psychometric findings for the balanced time perspective scale (BTPS). Can J Behav Sci. 2011;43(2):111–8.
8. Cáceda R, Moskovciak T, Prendes-Alvarez S, Wojas J, Engel A, Wilker SH, et al. Gender-specific effects of depression and suicidal ideation in prosocial behaviors. PLoS One. 2014;9(9):e108733.
9. Morin AJS, Moullec G, Maïano C, Layet L, Just JL, Ninot G. Psychometric properties of the Center for Epidemiologic Studies Depression Scale (CES-D) in French clinical and nonclinical adults. Rev Epidemiol Sante Publique. 2011;59(5):327–40.
10. Radloff LS. The CES-D Scale: A Self-Report Depression Scale for Research in the General Population. Appl Psychol Meas. 1977;1:385–401.
11. Weyant JM. Effects of mood states, costs, and benefits on helping. J Pers Soc Psychol. 1978;36:1169–76.
12. Greitemeyer T, Osswald S, Brauer M. Playing Prosocial Video Games Increases Empathy and Decreases Schadenfreude. Emotion. 2010;10(6):796–802.
13. Gaesser B, Keeler K, Young L. Moral imagination: Facilitating prosocial decision-making through scene imagery and theory of mind. Cognition. 2018;171:180–93.
14. Gaesser B, Shimura Y, Cikara M. Episodic simulation reduces intergroup bias in prosocial intentions and behavior. J Pers Soc Psychol [Internet]. 2020 Apr;118(4):683–705.
